# Supplementary material for: Influence of N-Acetyl-L-Cysteine on the Pharmacokinetics and Antibacterial Activity of Marbofloxacin in Chickens
Source: Antibiotics (Basel). 2025 Apr 10;14(4):393. doi: 10.3390/antibiotics14040393 (PMC12024055; doi:10.3390/antibiotics14040393)
Supplement: Supplementary file 1 [file antibiotics-14-00393-s001.zip › antibiotics-3549017-supplementary.pdf]

Table S1. Plasma concentrations of intravenously administered marbofloxacin at a dose rate of 5 mg/kg BW in healthy broiler chickens (n = 12)

| Time after first dose (h) | Concentration (μg×mL$^{-1}$) in healthy chickens, treated intravenously with marbofloxacin | | | | | |
|---|---|---|---|---|---|---|
| | Chicken 1 | Chicken 2 | Chicken 3 | Chicken 4 | Chicken 5 | Chicken 6 |
| 0.083 | 3.96 | 9.01 | 9.03 | 4.38 | 10.08 | 9.68 |
| 0.025 | 15.94 | 8.05 | 6.39 | 9.28 | 7.65 | 2.87 |
| 0.75 | 5.01 | 5.50 | 5.22 | 5.32 | 6.41 | 5.14 |
| 3 | 3.52 | 2.92 | 2.29 | 2.26 | 3.61 | 2.96 |
| 9 | 0.74 | 0.96 | 0.43 | 1.14 | 1.54 | 1.39 |
| 14 | 0.33 | 0.35 | 0.18 | 0.56 | 0.67 | 0.75 |
| 24 | 0.05 | 0.08 | 0.02 | 0.11 | 0.11 | 0.14 |
| | Concentration (μg×mL$^{-1}$) in healthy chickens, treated intravenously with marbofloxacin | | | | | |
| | Chicken 7 | Chicken 8 | Chicken 9 | Chicken 10 | Chicken 11 | Chicken 12 |
| 0.5 | 5.97 | 6.53 | 6.80 | 6.07 | 3.24 | 5.44 |
| 1 | 5.21 | 10.06 | 7.50 | 5.99 | 5.60 | 4.62 |
| 1.5 | 3.61 | 3.46 | 4.24 | 4.34 | 3.11 | 3.89 |
| 2 | 3.44 | 2.98 | 2.82 | 3.24 | 3.40 | 3.08 |
| 6 | 1.97 | 2.38 | 1.70 | 1.47 | 1.59 | 2.56 |
| 12 | 0.56 | 0.79 | 0.34 | 0.42 | 0.38 | 0.85 |
| 30 | 0.02 | 0.05 | 0.02 | 0.03 | 0.03 | 0.03 |

Table S2. Plasma concentrations of orally administered marbofloxacin at a dose rate of 5 mg/kg BW in healthy broiler chickens (n = 12)

| Time after first dose (h) | Concentration ($\mu g \times mL^{-1}$) in healthy chickens, treated orally with marbofloxacin | | | | | |
|---|---|---|---|---|---|---|
| | Chicken 1 | Chicken 2 | Chicken 3 | Chicken 4 | Chicken 5 | Chicken 6 |
| 0.083 | 0.65 | 0.88 | 0.11 | 0.16 | 0.79 | 0.57 |
| 0.025 | 1.82 | 1.14 | 0.38 | 0.65 | 1.65 | 1.10 |
| 1 | 5.01 | 4.66 | 1.77 | 0.84 | 2.01 | 1.92 |
| 1.5 | 4.70 | 4.48 | 1.64 | 2.24 | 2.95 | 3.43 |
| 3 | 3.46 | 2.80 | 2.14 | 3.34 | 2.52 | 3.14 |
| 8 | 1.82 | 1.52 | 1.58 | 2.03 | 1.79 | 1.78 |
| 12 | 0.41 | 0.53 | 0.53 | 0.68 | 0.64 | 0.51 |
| 24 | 0.08 | 0.04 | 0.06 | 0.03 | 0.06 | 0.05 |
| 36 | 0.03 | <LOQ | 0.02 | <LOQ | 0.01 | 0.01 |

| | Concentration ($\mu g \times mL^{-1}$) in healthy chickens, treated orally with marbofloxacin | | | | | |
|---|---|---|---|---|---|---|
| | Chicken 7 | Chicken 8 | Chicken 9 | Chicken 10 | Chicken 11 | Chicken 12 |
| 0.5 | 0.62 | 1.86 | 1.20 | 1.24 | 0.77 | 1.15 |
| 0.75 | 0.66 | 0.70 | 1.69 | 1.22 | 2.06 | 1.95 |
| 2 | 2.37 | 2.29 | 3.15 | 1.94 | 3.99 | 3.49 |
| 6 | 2.14 | 1.93 | 1.81 | 1.95 | 2.23 | 2.32 |
| 10 | 0.62 | 0.68 | 0.59 | 0.62 | 0.82 | 0.62 |
| 30 | <LOQ | 0.04 | <LOQ | 0.04 | 0.09 | 0.03 |

LOQ – limit of quantification

Table S3. Plasma concentrations after repeated oral administration of marbofloxacin at a dose of 5 mg/kg BW (♦) and after pre-treatment with N-acetyl-L-cysteine via the feed at a dose of 400 mg/kg BW. N-acetyl-L-cysteine administration started two days before the first oral dose of marbofloxacin (administered into the crop). The next doses of marbofloxacin were administered via the drinking water.

| Time after first dose (h) | Concentration (μg×mL$^{-1}$) in healthy chickens, treated orally with marbofloxacin via crop at a single dose of 5 mg/kg BW and with N-acetyl-L-cysteine via the feed at a dose of 400 mg/kg BW | | | | | |
|---|---|---|---|---|---|---|
| | Chicken 1 | Chicken 2 | Chicken 3 | Chicken 4 | Chicken 5 | Chicken 6 |
| 0.083 | 0.77 | 0.59 | 0.31 | 0.71 | 0.03 | 0.55 |
| 0.5 | 0.16 | 1.48 | 1.62 | 1.97 | 0.67 | 0.37 |
| 1 | 0.02 | 2.58 | 2.51 | 2.82 | 1.28 | 1.46 |
| 3 | 0.58 | 1.99 | 2.52 | 1.70 | 1.07 | 1.28 |
| 8 | 1.03 | 0.46 | 0.65 | 0.58 | 0.42 | 0.89 |
| 12 | 0.49 | 0.10 | 0.14 | 0.19 | 0.16 | 0.39 |
| 24 | 0.05 | 0.06 | 0.03 | 0.02 | 0.02 | 0.09 |
| | Concentration (μg×mL$^{-1}$) in healthy chickens, treated orally with marbofloxacin via crop at a single dose of 5 mg/kg BW and with N-acetyl-L-cysteine via the feed at a dose of 400 mg/kg BW | | | | | |
| | Chicken 7 | Chicken 8 | Chicken 9 | Chicken 10 | Chicken 11 | Chicken 12 |
| 0.025 | 0.36 | 0.06 | 0.85 | 0.67 | 0.08 | 0.18 |
| 0.75 | 0.19 | 1.07 | 2.03 | 1.81 | 1.71 | 1.31 |
| 1.5 | 0.61 | 2.34 | 2.29 | 2.05 | 2.66 | 2.56 |
| 2 | 0.63 | 2.57 | 2.56 | 2.37 | 2.24 | 2.61 |
| 6 | 1.08 | 1.24 | 0.78 | 0.78 | 0.87 | 0.82 |
| 10 | 0.40 | 0.36 | 0.21 | 0.30 | 0.21 | 0.36 |
| Time after first dose (h) | Concentration (μg×mL$^{-1}$) in healthy chickens, treated orally with marbofloxacin via drinking water for four consecutive days at a dose of 5 mg/kg BW and with N-acetyl-L-cysteine via the feed at a dose of 400 mg/kg BW | | | | | |
| | Chicken 1 | Chicken 2 | Chicken 3 | Chicken 4 | Chicken 5 | Chicken 6 |
| 36 | 0.32 | 0.50 | 0.35 | 0.55 | 0.46 | 0.73 |
| 48 | 0.28 | 0.38 | 0.37 | 0.34 | 0.33 | 0.81 |
| 96 | 0.20 | 0.45 | 0.32 | 0.38 | 0.31 | 0.58 |
| 120 | 0.20 | 0.17 | 0.31 | 0.27 | 0.36 | 0.56 |
| 126 | 0.13 | 0.05 | 0.05 | 0.05 | 0.16 | 0.28 |
| 132 | 0.06 | 0.06 | 0.06 | 0.12 | 0.12 | 0.20 |
| | Concentration (μg×mL$^{-1}$) in healthy chickens, treated orally with marbofloxacin via drinking water for four consecutive days at a dose of 5 mg/kg BW and with N-acetyl-L-cysteine via the feed at a dose of 400 mg/kg BW | | | | | |
| | Chicken 7 | Chicken 8 | Chicken 9 | Chicken 10 | Chicken 11 | Chicken 12 |
| 30 | 0.255 | 0.467 | 0.260 | 0.305 | 0.401 | 0.462 |
| 122 | 0.196 | 0.403 | 0.172 | 0.202 | 0.254 | 0.203 |
| 124 | 0.102 | 0.188 | 0.087 | 0.109 | 0.120 | 0.119 |
| 128 | 0.083 | 0.223 | 0.055 | 0.079 | 0.096 | 0.117 |
| 144 | 0.026 | <LOQ | <LOQ | <LOQ | 0.032 | 0.050 |
| 150 | <LOQ | <LOQ | 0.014 | <LOQ | <LOQ | <LOQ |

LOQ – limit of quantification
